# Postfabrication Tuning of Circular Bragg Resonators for Enhanced Emitter-Cavity Coupling

**DOI:** 10.1021/acsphotonics.3c01480

**Published:** 2024-01-19

**Authors:** Tobias M. Krieger, Christian Weidinger, Thomas Oberleitner, Gabriel Undeutsch, Michele B. Rota, Naser Tajik, Maximilian Aigner, Quirin Buchinger, Christian Schimpf, Ailton J. Garcia, Saimon F. Covre da Silva, Sven Höfling, Tobias Huber-Loyola, Rinaldo Trotta, Armando Rastelli

**Affiliations:** † Institute of Semiconductor and Solid State Physics, Johannes Kepler University Linz, Altenberger Straße 69, 4040 Linz, Austria; ‡ Dipartimento di Fisica, Sapienza University of Rome, Piazzale Aldo Moro 5, 00185 Rome, Italy; § Lehrstuhl für Technische Physik, Physikalisches Institut, Julius-Maximilians-Universität Würzburg, Am Hubland, 97074 Würzburg, Germany

**Keywords:** quantum dot, single photon, Purcell, digital etching, bullseye, Fano

## Abstract

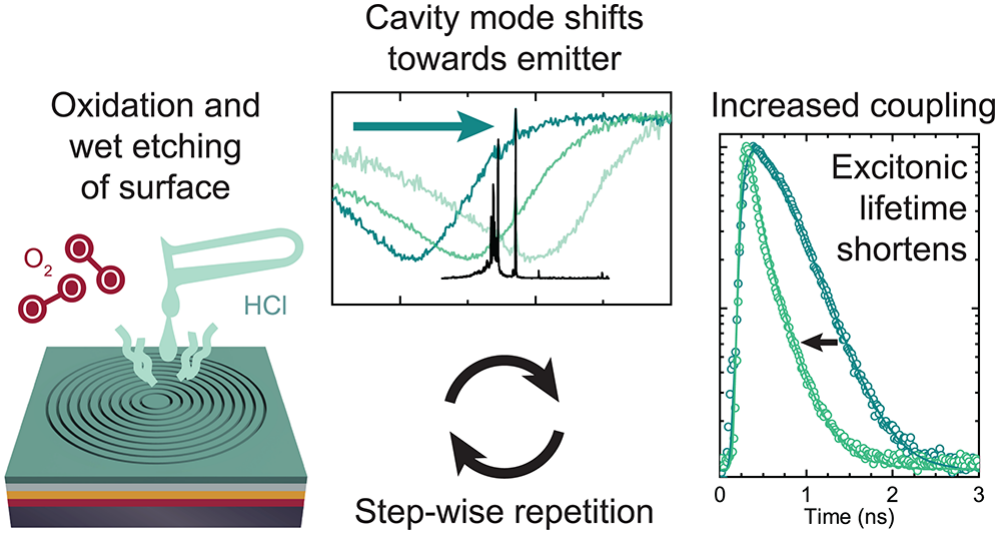

Solid-state quantum emitters embedded in circular Bragg resonators are attractive due to their ability to emit quantum light with high brightness and low multiphoton probability. As for any emitter-microcavity system, fabrication imperfections limit the spatial and spectral overlap of the emitter with the cavity mode, thus limiting their coupling strength. Here, we show that an initial spectral mismatch can be corrected after device fabrication by repeated wet chemical etching steps. We demonstrate an ∼16 nm wavelength tuning for optical modes in AlGaAs resonators on oxide, leading to a 4-fold Purcell enhancement of the emission of single embedded GaAs quantum dots. Numerical calculations reproduce the observations and suggest that the achievable performance of the resonator is only marginally affected in the explored tuning range. We expect the method to be applicable also to circular Bragg resonators based on other material platforms, thus increasing the device yield of cavity-enhanced solid-state quantum emitters.

## Introduction

Bright sources of nonclassical light play a crucial role in the development of quantum technologies such as quantum communication and quantum information processing.^1−3^ Over the past decades, various schemes for generating single photons^4^ and entangled photon pairs^5^ have emerged. Among these schemes, sources based on spontaneous parametric down conversion are commonly used for heralded single photons and entangled photon pairs with a near-unity degree of entanglement.^6^ However, the stochastic nature of photon generation of such sources imposes fundamental limitations on their maximum brightness.^7^

Solid-state emitters are promising alternatives for single photon and entangled photon sources^8^ as they combine optical quality similar to atomic emission^9−11^ with compact nanoscale integration, enabling the use of established manufacturing processes of the host system.^12^ Nevertheless, solid-state systems present challenges, including an inhomogeneous distribution of emission properties among multiple emitters, as well as homogeneous and inhomogeneous broadening of the emission line width, which reduces photon indistinguishability. Moreover, for host materials with a large refractive index, the extraction efficiency of photons from the solid-state is limited due to total internal reflection.

To address these challenges, circular Bragg grating resonators (CBRs), also known as “bullseye cavities” or “bullseye antennas,”^13^ emerged as very appealing structures enabling high extraction efficiency over a large frequency range and Purcell enhancement of quantum emitters coupled to optical resonator modes. The bullseye design has been successfully implemented in various emitter systems, including III–V semiconductor quantum dots (QDs),^14−21^ colloidal QDs,^28,29^ emitters in GaN layers,^22^ emitters in WSe_2_ monolayers,^27^ color centers in hexagonal boron nitride,^23^ and N and Si vacancy centers in diamond.^24−26^

Although the low quality factor of the CBR allows some tolerance to spectrally match the cavity mode (CM) with integrated emitters, the realization of such devices remains challenging. Fabrication imperfections limit the accuracy of the spatial and spectral overlap between the emitter and desired CM, especially for short-wavelength emitters embedded in high-refractive-index materials, where deviations of a few nanometers lead to CM shifts comparable to the CM spectral width. This results in a low yield of deterministically patterned devices, while still requiring time-consuming precharacterization of the emitters. Several tuning approaches can be employed to match the emission energy of an emitter with a CM. Tuning the emission energy of a quantum emitter by the application of elastic stress^21,30−33^ offers a wide tuning range, has a fine resolution, and neither influences the optical quality nor the low multiphoton contribution. However, strain-tuning simultaneously shifts the CM as well^30^ and requires additional sample fabrication steps. Electric fields can be used to shift the emitter transition energies without appreciably affecting the CM position.^34,35^ The integration is similarly challenging but can improve both the optical quality of the emission and suppress blinking of the single photon source due to deterministic charge control.^36,37^ The maximum demonstrated tuning range, however, is limited to ∼5 meV (∼2.5 nm). An even smaller tuning range can be expected if the electric field is used also to remove source blinking. A different approach to increase emitter-cavity coupling involves tuning the CM instead of the emitter, which is comparably simpler to realize. Free-carrier injection^38^ can be employed to tune the CM, utilizing a nonlinear effect to alter the refractive index with a fast control laser pulse, allowing for a moderate blue shift of a meV. The method’s limits, however, are the small tuning range, the strong dependence of the effect on the geometry of the resonator and an unclear influence on the optical quality of the emission. Another laser-based approach is local laser-assisted temperature tuning,^39^ which can be used to fine-tune the emission of a quantum emitter. Similar to strain-tuning, the CM position is affected. In addition, the emission suffers from line width broadening, limiting the maximum tuning range to less than a meV. To avoid this degradation, one can employ laser-assisted oxidation of the surface.^40,41^ Due to local heating of the surface being exposed to air, oxidation is accelerated, leading to a permanent change in the geometry of single cavities also in the absence of the laser, typically resulting in a blue shift of CMs. The demonstrated tuning range of ∼12 meV (∼6 nm) is limited due to the decreasing efficiency of the oxide growth with increasing layer thickness. Using atomic layer deposition of an oxide layer,^42^ CMs can be red-shifted permanently up to tens of meV (tens of nm), mainly limited by the resonator geometry. This method also provides an additional passivating layer, which may benefit the optical quality of the emission.^43^ Alternatively, gas condensation at low temperatures^39,44^ can be employed to red-shift CMs utilizing a controlled injection of Xe or N atoms into an evacuated cryostat. This technique allows for a demonstrated red shift of CMs of up to ∼10 meV (∼5 nm) and can be reversed by heating the sample above the sublimation temperature of the used gas. To realize a blue shift of CMs, repeated wet chemical etching^45,46^ allows for broadband tuning over tens of meV (tens of nm) with a fine resolution. Still, concerns may arise about possible degradation of the optical performance of the emitters and resonators and about the structure integrity due to etching of the dielectric layer placed usually between the CBR and backside reflector.^15,16^

In this study, we demonstrate that repeated wet chemical etching of the native oxides of GaAs and AlGaAs enables postfabrication tuning of the CM wavelength by 31(3) meV (16(2) nm), resulting in spectral matching with the emission of an embedded GaAs QD obtained by droplet-etching epitaxy.^47^ This tuning approach enables a 4-fold Purcell enhancement, here limited by inaccurate spatial overlap between the emitter and cavity, without compromising either the low multiphoton contribution, optical quality, or the structural integrity of the AlGaAs resonator on Al_2_O_3_ dielectric layer.

## Concept and Simulation

**Figure 1:**
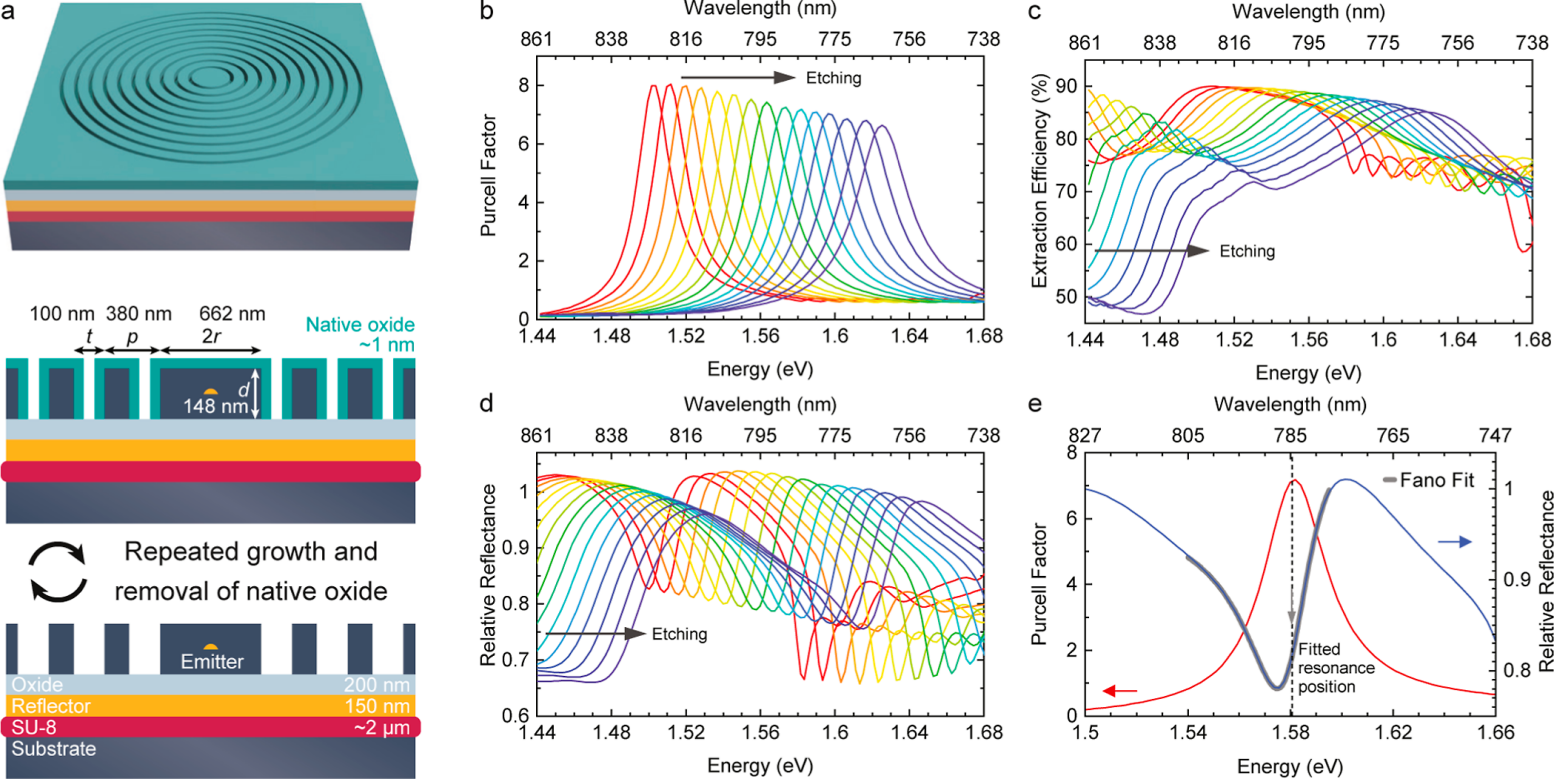
(a) Sketch representing the CBR structure with geometric parameters before processing and the repeated removal of the native oxide by wet chemical etching. FDTD simulation results of the (b) Purcell enhancement, (c) extraction efficiency, and (d) CBR reflectance relative to that of the surrounding planar areas for repeated etch steps as a function of the photon energy and wavelength. (e) Reflectance curve fitted with a Fano line shape with marked resonance position *E*_c_ compared to the corresponding spectrum of the Purcell factor.

The sample under investigation, as sketched in Figure 1a, consists of a 140 nm thick Al_0.33_Ga_0.67_As membrane, containing a GaAs QD layer in the center, with 4 nm thick GaAs capping layers on the top and bottom and a back-reflector underneath. CBRs with three designs (d1, d2, and d3), each characterized by a different radius *r* of the central disc, are dry-etched deterministically on QD sites, exposing the side walls of Al_0.33_Ga_0.67_As to the ambience. Details of sample fabrication are provided in the Supporting Information. CBR structures on this sample exhibit limited spatial and spectral overlap with the QDs. The QD emission displays a degree of polarization higher than 60%, which we attribute to a misplacement of the QDs from the center of the cavity.^48^ Furthermore, no Purcell enhancement was observable after fabrication due to a systematical blue shift of about 26–49 meV (13–25 nm) compared to the CM (see below).

We now illustrate the concept of mode tuning by using finite-difference time-domain (FDTD) simulations for the CBR sketched in Figure 1a with an emitting dipole located at its center. (For details, cf. to the section “Simulation” in the Supporting Information.) Figure 1b–d illustrates that by material removal from the CBR, i.e., by reducing the center disc radius *r*, decreasing the membrane thickness *d*, and increasing the trench width *t*, in steps of 1.5 nm (in total 21 nm), we expect a blue shift in the spectral position of the maximum Purcell factor (Figure 1b). Simultaneously, the extraction efficiency peak (Figure 1c) is also blue-shifted. The peak value of the extraction efficiency slowly decreases for increasing etch steps as the structure continuously departs from the optimized design for a given wavelength (see also simulations of the quality factor in the Supporting Information). Nevertheless, the efficiency stays ≳85% in a ∼20–40 meV (10–20 nm) wide range around the CM position, so we can concentrate on shifting the CM position to achieve Purcell enhancement without worrying about significant intensity drops.

In the experiment, it is convenient to use reflectance spectroscopy to obtain the spectral position of the CM. To unambiguously find the spectral position of the Purcell enhancement-maximum, we simulate the reflectance spectrum, as shown in Figure 1d. The asymmetry of the resulting reflectance dip arises from the Fano interference effect, which occurs in the presence of two possible pathways of scattering events from a discrete state and from a continuum.^49,50^ Light that is directly scattered from the surface interferes with light that scatters resonantly coupled to the CM.^51^ This effect is also observable in CBRs.^52^ The Fano line shape *I*(*E*) is given by
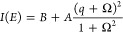
1with energy *E* and constants *A*, *B*, and Ω = 2(*E* – *E*_c_)/Γ_c_, where *E*_c_ is the CM position and Γ_c_ the resonance line width. The Fano parameter *q* is the ratio of direct and resonant transition amplitudes of scattering events^49^ and influences the asymmetry of lineshapes, converging to a Lorentzian line shape for *q* = 0. Fitting the resonance line shape of the simulated reflectance spectrum with eq 1 reveals that *E*_c_ matches the position of maximum Purcell enhancement well, as can be seen in Figure 1e.

## Results and Discussion

Experimental results are obtained by monitoring the properties of different CBRs and embedded QDs upon repeated etch cycles of native oxide. The excitonic QD emission is centered at 1.581(8) eV (784(4) nm), whereas the CM positions before etching are 1.548(4) meV (801(2) nm) for d1-CBRs, 1.542 meV (804(2) nm) for d2-CBRs, and 1.538 meV (806(2) nm) for d3-CBRs. One etch cycle consists of the growth of native oxide,^53,54^ where the oxide thickness is dependent on the time of exposure to the ambience, followed by a self-limited removal of that oxide by soaking the sample for 1 min in 18.5% HCl.^55^

**Figure 2:**
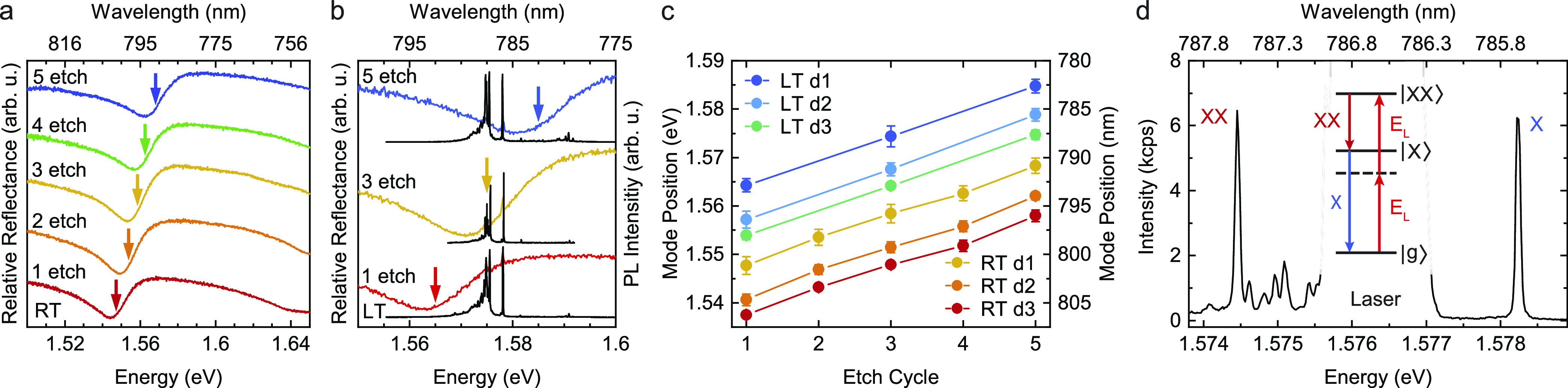
Relative reflectance spectra of a representative CBR, showing the etch-induced blue shift of the CM at (a) RT and (b) LT (colored curves). Resonance positions *E*_c_ are marked with an arrow. (b) also shows the PL spectrum (black curves) of the embedded QD obtained under above-bandgap excitation. (c) Mean values of *E*_c_ for 10 CBRs having the same nominal design (either d1, d2, or d3) at RT and LT as a function of the number of performed etch cycles. Error bars correspond to the standard deviation. (d) TPE spectrum with labeled X and XX transitions and the remaining excitation laser in the background. Inset: Level scheme of the |XX⟩ population and radiative cascade decay.

Figure 2a shows the ratio of the reflected signal of an incident focused beam of thermal light on a representative CBR and on the surrounding planar regions, i.e., the relative reflectance, after each of the 5 performed etch cycles at room temperature (RT). All reflectance spectra are fitted with a Fano line shape, and the resonance positions *E*_c_ are marked with an arrow. In order to quantify the mismatch of the emission to the CM, we record both the photoluminescence (PL) of the contained QD under above-band gap excitation and the relative reflectance spectra at low temperature (LT), ∼10 K (see Figure 2b). Due to cooling the resonators to LT, a blue shift of the resonance position of 16.4(3) meV (8.4(2) nm) is observed. The spectral position of the QD emission is not affected by the etching. Figure 2c shows the mean values of the resonance positions of ten structures with the same nominal design (either d1, d2, or d3) for each etch cycle, revealing an average CM blue shift of 5.1(2) meV (2.6(1) nm) per etch cycle. The exposure time to ambience influences the amount of native oxide forming on the semiconductor surface and consequently the magnitude of the CM shift unlike the soaking time in HCl, as the oxide etching is self-limited.^46^ The shift induced by the first etch cycle is more than twice as large as the average, as the time between the sample fabrication and the first etching was on the order of several days, whereas the exposure time of etch cycles 1, 2, 3, and 4 is ∼3 h. The CM shift induced by the fifth etch cycle is also larger than the average, i.e., 6.1 meV (3.1(2) nm), since the exposure to ambience lasted one full day. We expect the opposite behavior and, therefore, fine-tuning of ∼1 nm per etch cycle when exposing surfaces for tens of minutes to air.^46^ The overall explored tuning range shows a total CM blue shift of 31(3) meV (16(2) nm). From a comparison between the measured and calculated shift based on the simulations, we estimate that each etch step results in the removal of 0.9(3) nm of material from each surface.

To analyze changes in the emission characteristics of on-demand emitters in tuned CBR structures, we employ a two-photon excitation (TPE) scheme, allowing us to access the decay times (lifetimes) of the biexciton |XX⟩ and exciton |X⟩ states in parallel. The biexciton level |XX⟩ is resonantly excited with a pulsed laser with a repetition rate of 80 MHz, where the laser energy *E*_L_ is set to half of the energy difference between |XX⟩ and ground state |g⟩ (*E*_XX_) and with the laser power adjusted to maximize the |XX⟩ population.^56^ An additional above-bandgap light source is used to maximize the population efficiency and reduce QD blinking.^57^ A spectrum under such excitation conditions is provided in Figure 2d, showing the transitions from |XX⟩ to |X⟩ (XX photons) and from |X⟩ to |g⟩ (X photons) in a cascade process.

**Figure 3:**
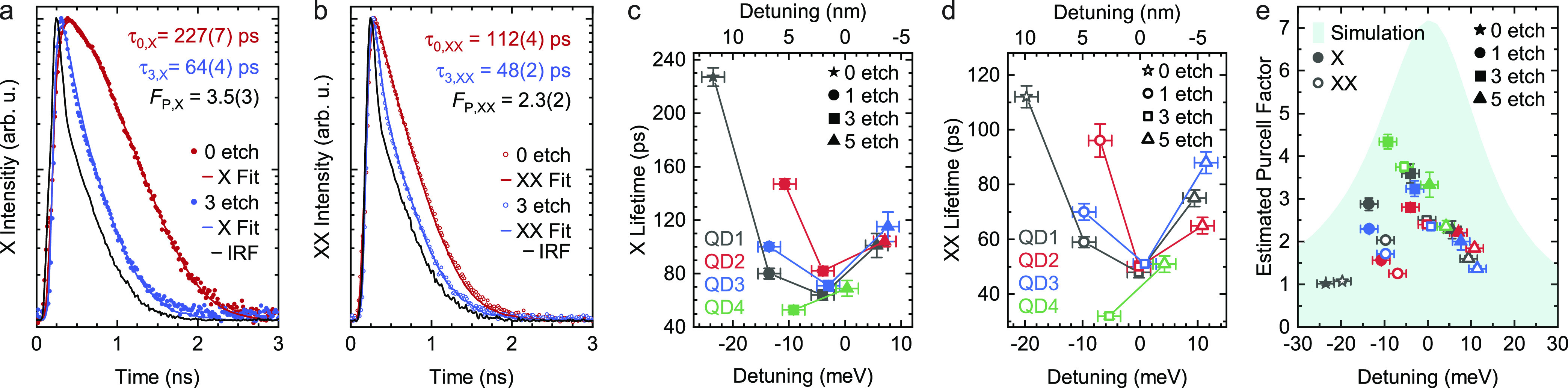
Time-correlated single-photon-counting measurements of X (a) and XX (b) photons emitted by QD1 upon TPE before (0 etch) and after 3 etch cycles. IRF denotes the instrument response function, and the quoted lifetimes clearly indicate a lifetime reduction, which we attribute to the Purcell effect. (c) Analysis of lifetime values as a function of detuning from the CM and different etch cycles for X and (d) XX. (e) Estimated Purcell factor as a function of detuning and simulated Purcell factor spectrum for a QD placed in the resonator center. (c–e) Different colors are used for different QDs, as labeled in (c,d), while full/empty symbols are used for X/XX photons. Error bars (vertical) of the estimated Purcell factor are based only on the uncertainty on the corresponding lifetime measurements. Error bars (horizontal) in the detuning axis are 2 meV, estimated experimentally from reflectance measurements. The detuning values of the star-data points were not directly measured but are estimations from the CM shift between etch cycles 0 and 1, measured using another CBR.

From Figure 2b, we expect the highest coupling between an exciton confined in QD1 and the corresponding CM after etch cycle 3. To confirm this, we performed time-correlated single-photon-counting measurements of X [XX] photons emitted by QD1, as displayed in Figure 3a,b. By comparing the decay dynamics of the untreated sample and after the third etch cycle, we observe a reduction of the lifetime τ for the |X⟩ [|XX⟩] states and estimate a Purcell enhancement *F*_P_ of 3.5(3) [2.3(2)] based on *F*_P_ = τ_0_/τ_3_ where the index denotes the number of performed etch cycles. Lifetime values τ_0_ of |X⟩ and |XX⟩ of QDs inside as-fabricated cavities coincide with values from similar QDs in the bulk (i.e., 230 ps for X and 120 ps for XX), as expected from the simulation, yielding *F*_P_ ∼ 1 for such detuning. We extend the study on decay dynamics to 4 QDs and present values for τ of X [XX] photons in Figure 3c [Figure 3d] as a function of detuning of the transition with the CM position, i.e., (*E*_c_ – *E*_X/XX_). A clear reduction of the lifetime of all studied QDs is visible when approaching low detuning, followed by an increase when the CM is further blue-detuned. In order to compare the expected Purcell enhancement with the experimental data, we estimate the *F*_P_ for the X and XX photons using the typically measured lifetime values in bulk, which we quoted above. The results for all measured QD/CBR systems are plotted in Figure 3e as a function of detuning. The highest Purcell factor we observed is 4.3(2). In spite of the fact that the measured and simulated cavity quality factors match well (see the Supporting Information), the simulation predicts Purcell factors that are consistently higher than those extracted from the experiment (see shaded curve in Figure 3e). We attribute this observation and the pronounced scatter of the data points to the already mentioned spatial mismatch between emitters and the cavity, since a radial misplacement exceeding ∼35 nm leads to a suboptimal coupling even in case of spectral matching.^58^

**Figure 4:**
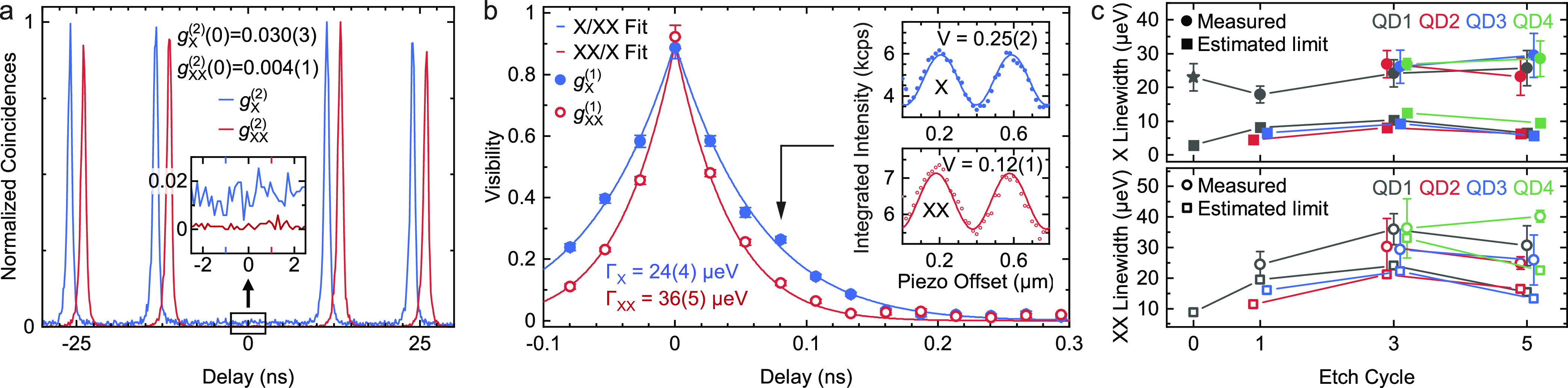
(a) *g*^(2)^(*t*) autocorrelation and (b) *g*^(1)^(*t*) coherence measurements of X (blue line) and XX (red line) photons for QD1 after (a) 5 and (b) 3 etch cycles. (a) XX/X peaks are horizontally shifted by ±1 ns for ease of reading. (b) Insets show X and XX interference fringes at a 80 ps time delay. (c) X (full/solid) and XX (empty/dashed) transition line width values of the studied QDs for different etch cycles comparing measured line widths (circle) to the expected natural line widths (square) based on the respective lifetimes. The star-data point was obtained under nonresonant excitation.

To probe the effect of etching on the multiphoton emission probability, we perform a Hanbury Brown–Twiss experiment after each etch cycle to measure the autocorrelation function *g*^(2)^(*t*) for time delays *t* and evaluate it at *t* = 0. The experimental results for the last etch cycle of QD1 are provided in Figure 4a, yielding *g*_X_^(2)^(0) = 0.030(3) and *g*_XX_^(2)^(0) = 0.004(1). A strong suppression of the peaks at 0-time delay proves the single photon generation. As depicted in the zoomed-in panel, the *g*_X_^(2)^(0) value of the X photons is somewhat higher than for XX photons, which we attribute to the unintentional population of the |X⟩ state by the continuous-wave nonresonant laser used to reduce blinking. The *g*^(2)^(0) values for X and XX photons of QD4, which have the shortest lifetimes of the measured structures, are *g*_X_^(2)^(0) = 0.029(2) and *g*_XX_^(2)^(0) = 0.009(6), showing that the accelerated decay does not lead to re-excitation.^21,59^ Measurements of the autocorrelation function for both X and XX photons on 4 different QDs after etch cycles 1, 3, and 5 show that the values at 0-time delay are not affected by the etching (see section “Autocorrelation Data” in the Supporting Information).

To gain further insights into the possible degradation of the optical quality of X and XX photons, we perform Michelson interferometry measurements to obtain the first-order coherence function, *g*^(1)^(*t*), by probing the visibility of interference fringes for different time delays *t* between the optical paths of the interferometer, as depicted in Figure 4b. The potential influence of etching on line width broadening is determined by repeating *g*^(1)^(*t*) measurements on more QDs after different etch cycles, focusing on cycles 3 and 5, where we expect the strongest emitter-cavity coupling. In Figure 4c the obtained line width values are shown and compared to the estimated natural (lifetime-limited) line width in a cascade process,^60^ i.e., ℏ/τ_X_ for |X⟩, and ℏ/τ_XX_ + ℏ/τ_X_ for |XX⟩. The measurements indicate that no pronounced broadening of the line width can be observed within the error bars, even when applying 5 etch cycles, rendering this postfabrication tuning method effective for broad-band tuning without deteriorating the optical quality of single photons.

## Conclusions

In summary, we have demonstrated that repeated wet-chemical etching and air exposure provide a simple and effective method to blue-shift the CMs of circular Bragg grating resonators in a spectral range of at least 31(3) meV (16(2) nm). Furthermore, this postfabrication tuning allows one to increase the emitter-cavity coupling in initially detuned systems, leaving the low multiphoton emission probability as well as the high optical quality practically unaffected. The highest value of Purcell enhancement that could be obtained within this study is *F*_P_ = 4.3(2), resulting in an excitonic lifetime of 53(2) ps and a line width of 2.2(2) times the Fourier transform limit. In our experiment, we expect the value of *F*_P_ to be mostly limited by a misplacement of the QDs from the center of the cavity, resulting from an error during fabrication and causing a partially polarized emission.

In principle, the investigated technique can be used to tune resonators in any solid-state emitter system that grows a native oxide, both with broad-range and with fine resolution, utilizing the ambient-exposure time between etch cycles to control a single CM shift. Compared to other tuning methods mentioned in the introduction, only repeated wet-chemical etching enables a substantial blue shift of CMs, compromising neither the optical quality nor the low multiphoton contribution of the QDs, while simultaneously being simpler to implement, especially in contrast to tuning with strain fields^21,30−33^ or electric fields.^34,37,39^ If a subsequent red shift of the CMs is desired, a combination with, for instance, atomic layer deposition of an oxide^42^ promises to benefit also the optical quality.^43^ We expect this work to be beneficial to the community, serving as a tool to increase the yield of working samples and, therefore, accelerate research on solid-state quantum emitters as resources in novel quantum technologies.

## Data Availability

The data underlying this study are openly available in Zenodo at https://doi.org/10.5281/zenodo.10461467.

## Supporting Information Available

Complementary data (quality factor, polarization of CMs, gas condensation, and *g*^(2)^(0) values) and methods (simulation, sample fabrication, measurement setup, and details of optical characterization) (PDF)
