# Supplementary material for: Postfabrication Tuning of Circular Bragg Resonators for Enhanced Emitter-Cavity Coupling
Source: ACS Photonics. 2024 Jan 19;11(2):596–603. doi: 10.1021/acsphotonics.3c01480 (PMC10885778; doi:10.1021/acsphotonics.3c01480)
Supplement: Supplementary file 1 — ph3c01480_si_001.pdf [file ph3c01480_si_001.pdf]

Supporting Information: Postfabrication Tuning of Circular Bragg Resonators for Enhanced Emitter-Cavity Coupling

Tobias M. Krieger,*,† Christian Weidinger,† Thomas Oberleitner,† Gabriel Undeutsch,† Michele B. Rota,‡ Naser Tajik,† Maximilian Aigner,† Quirin Buchinger,¶ Christian Schimpf,† Ailton J. Garcia Jr.,† Saimon F. Covre da Silva,† Sven Höfling,¶ Tobias Huber-Loyola,¶ Rinaldo Trotta,‡ and Armando Rastelli*,†

†*Institute of Semiconductor and Solid State Physics, Johannes Kepler University Linz, Altenberger Straße 69, 4040 Linz, Austria*

‡*Dipartimento di Fisica, Sapienza University of Rome, Piazzale Aldo Moro 5, 00185 Rome, Italy*

¶*Lehrstuhl für Technische Physik, Physikalisches Institut, Julius-Maximilians-Universität Würzburg, Am Hubland, 97074 Würzburg, Germany*

E-mail: tobias.krieger@jku.at; armando.rastelli@jku.at

1 Complementary Data

1.1 Quality Factor

From the analysis of cavity mode (CM) positions upon repeated etching of circular Bragg grating resonators (CBRs), as discussed in the main text, the quality factor Q can be extracted as well. Q is defined as $Q = E_c/\Gamma_c$ with E_c being the CM position, and Γ_c the full

width at half maximum (FWHM) of the resonance. Figure S1 shows the mean values of Q, differentiating between CBR designs d1, d2, and d3, low temperature (LT), and room temperature (RT), corresponding to the same CBRs analyzed in Figure 2(c) in the main text. In the experiment we see a slight decrease of Q values for increasing etch cycles, which is fully consistent with the calculation results. To compare measured Q values with the simulation, the abscissae require to be matched with each other. As it was already presented in the main text, the material removal depth used in the simulation is 1.5 nm per etching, whereas the experimentally obtained value is 0.9 nm per etch cycle. A linear fit of the measured values yields a decrease of Q of 2.6(4) per etch cycle.

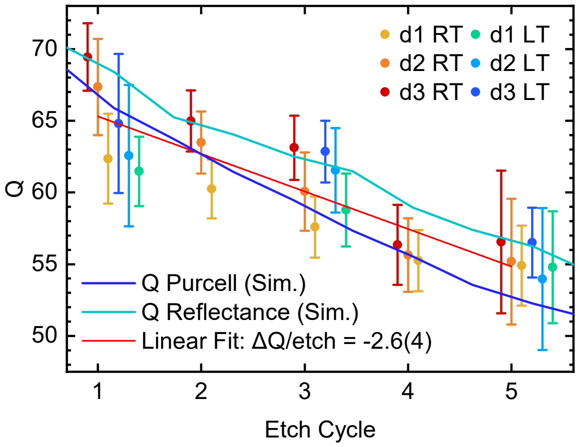

Figure S1: Measured Q factor as function of etch cycles, compared to values extracted from the simulation. Error bars are the standard deviation including a systematic error of ± 2, found from the uncertainty of Q resulting from fitting reflectance spectra with different fit ranges. Data points are shifted horizontally around the respective etch cycle for better clarity.

1.2 Polarization-Resolved Measurements

Polarization-resolved measurements of light reflected from the cavity, as well as photoluminescence (PL) signal from measured quantum dots (QDs) is performed by turning a half-wave plate in front of a polarizer step-wise by the angle $\Delta\theta$ in a range of 180°, recording a spectrum after each turning step. Polarized features in such a spectrum series are oscillating as function of 2θ. As mentioned in the main text, QDs exhibit polarized emission, where the

majority shows a polarization degree between 60 % and 99 % and a common polarization axis within 8° standard deviation.

We also measure a polarization-splitting of horizontal (H) and vertical (V) resonator modes with a magnitude of about 1 nm (1.9 meV) among the studied CBRs. Relative reflectance spectra of such modes of a representative CBR are given in Figure S2. Due to the large overlap of H and V modes, we attribute no substantial effect of this splitting on the polarized emission of the QD.

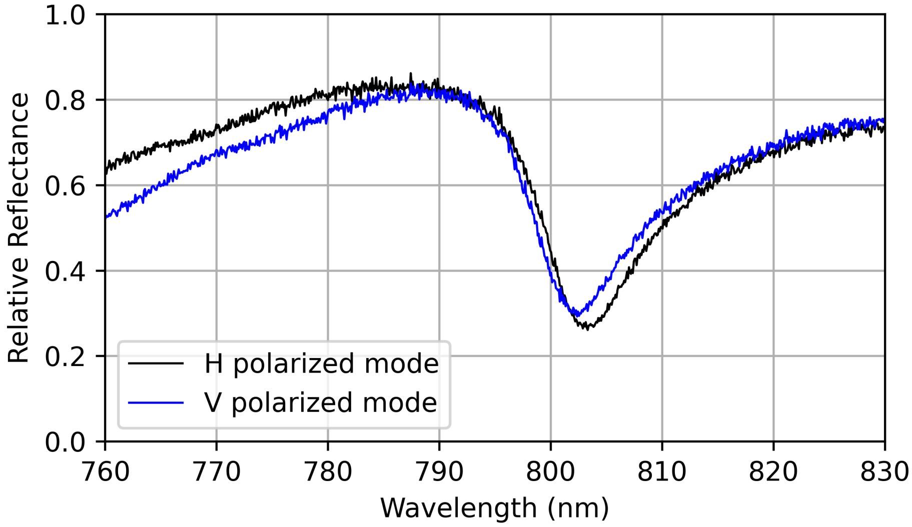

Figure S2: Relative reflectance of H and V polarized modes shifted 1 nm (1.9 meV) with respect to each other. The resonance position of the H polarized mode is at 801.7 nm (1.5465 eV), whereas the V polarized mode is located at 800.7 nm (1.5484 eV).

1.3 Gas Condensation

When measuring CM positions of CBRs at low temperature (< 8 K) for etch cycle 3, the CM did not shift as anticipated. While we were expecting a temperature-induced blue-shift of 8.4(2) nm (as seen in the main text), we observed a smaller blue-shift of 6(2) nm instead. The pressure during this measurement was $\sim 10^{-4}$ mbar, compared to a value $< 10^{-6}$ mbar that we usually achieve. After careful inspection of the vacuum system, the experiment was repeated on another day with a pressure of $< 10^{-6}$ mbar, resulting in a temperature-induced

blue-shift of the CM of 8.2(2) nm. The difference of the CM positions between the first and second measurement (different pressure in the cryostat) at low temperature is 3(2) nm, whereas the CMs do not show any shift at room temperature. We attribute the observed behaviour to gas condensation on the CBRs.[S1] Another indicator of gas condensation on the sample surface is the observation of an increasing amount of visible contamination at low temperature at a pressure of $\sim 10^{-3}$ mbar. Dark spots appear and cover the sample surface and can be removed by heating the sample to room temperature. An image of such contamination is given in Figure S3. (Note that the quoted pressure values were measured in proximity to a turbomolecular pump, connected to the cryostat with an approximately 2.5 m long tube)

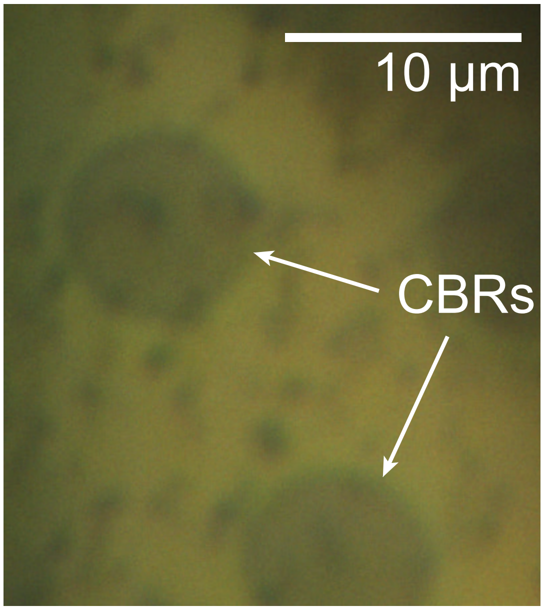

Figure S3: A wide-field image of the sample surface at a pressure of $\sim 10^{-3}$ mbar, showing CBRs and dark spots distributed over the sample.

1.4 Autocorrelation Data

The measured data set of values of $g^2(t)$ at 0-time delay for different etch cycles of the studied QDs is provided for X and XX photons in Table S1. Due to fast blinking dynamics of the source, only the first neighboring peaks are taken into account for normalization. The

used bin-width is 2 ns. Increased values of $g^{(2)}(0)$ of X photons are due to a background originating from non-resonant excitation, as discussed in the main text, and do not correlate with the repetition of etch cycles. Details about the measurement are given below in the subsection "Autocorrelation".

Table S1: Values of $g^{(2)}(0)$ for different etch steps. The highlighted row in green corresponds to the measurement given in the main text in the inset of Figure 4(a) and the one in teal corresponds to the QD with the shortest lifetimes of $|X\rangle$ and $|XX\rangle$.

QD No.	Etch Cycle	$g^{(2)}(0)_X$	$g^{(2)}(0)_{XX}$
1	0	0.059(6)	-
1	1	0.024(2)	0.025(3)
1	3	0.072(2)	0.007(1)
1	5	0.030(3)	0.004(1)
2	3	0.051(4)	0.012(2)
2	5	0.093(9)	0.006(2)
3	3	0.017(2)	0.005(1)
3	5	0.096(5)	0.049(4)
4	3	0.029(2)	0.009(6)
4	5	0.073(4)	0.014(2)

2 Methods

2.1 Simulation

The 3D finite-difference time-domain (FDTD) simulations are performed using the commercial solution ANSYS Lumerical. The CBR is centered in the simulation domain with the parameters periodicity p = 380 nm, trench width t = 100 nm, central disc radius r = 333 nm, membrane thickness d = 148 nm, and oxide thickness d = 200 nm, stacked on a gold substrate. The used refractive index of the membrane is $n_{\mathrm{AlGaAs}} = 3.3$ and of the oxide $n_{\mathrm{Al_2O_3}} = 1.64$. The implemented refractive index of gold is wavelength-dependent.[S2] For each simulated etch cycle, 1.5 nm of material is removed, i.e., t [d and r] increases [decreases] 1.5 nm per step.

The relative reflectance is defined as the division of the reflectance of the CBR by the

reflectance of the layer stack without CBR, to resemble the experiment. Therefore, in the simulations, a Gaussian beam with beam radius of 750 nm and a divergence angle of 30°is focused on the center of the CBR. The beam central wavelength is 800 nm with a range of 200 nm. The reflected intensities are recorded with a numerical aperture of 0.65. As the polarization of the Gaussian source is oriented along the x-axis, simulation time is reduced by using antisymmetric perfectly matched layer (PML) boundary conditions (BCs) normal to the x-axis and symmetric PML BCs normal to the y-axis. Due to the bottom gold mirror, metal BCs are used at the bottom of the simulation domain, whereas PML BCs are used at the top.

For the simulations targeting extraction efficiency and Purcell factor, a dipole source with a central emission wavelength of 780 nm and a range of 160 nm is used. The dipole emitter is oriented along the x-axis, allowing the above-mentioned symmetry and BCs to be used again. The extraction efficiency in the far-field can be calculated from a monitor placed above the structure in extraction direction.[S3] For this

$$\eta_{ex}(\theta) = \frac{\int_0^{2\pi} \int_0^{\theta} |E_\lambda(\theta', \phi)|^2 \sin(\theta') \mathrm{d}\theta' \mathrm{d}\phi}{\int_0^{2\pi} \int_0^{\pi/2} |E_\lambda(\theta', \phi)|^2 \sin(\theta') \mathrm{d}\theta' \mathrm{d}\phi} \cdot \frac{T}{F_\mathrm{P}}, \tag{1}$$

can be used, where E_λ is the electric far-field depending on the wavelength λ, F_P the Purcell factor and T the near-field transmittance, integrated over spherical coordinates θ, and ϕ.

The Purcell factor is calculated by the relation[S4]

$$\frac{\Gamma}{\Gamma_0} = \frac{P}{P_0} = F_\mathrm{P}, \tag{2}$$

where P is the enhanced energy dissipation derived from Poynting's theorem, Γ is the enhanced transition rate and P_0 and Γ_0 are the respective reference values of a source in bulk. Consequently, the Purcell factor is obtained from the simulations by placing monitors around the source which detect the transmission of radiated source power.

2.2 Sample Fabrication

QD samples used within this study were grown by molecular beam epitaxy (MBE). To assist the subsequent resonator fabrication, first, a 400 nm thick etch stop layer of $Al_{0.75}Ga_{0.25}As$ is grown on top of a commercial GaAs(001) wafer following GaAs-buffer growth. Then, a 4 nm GaAs layer and a 70 nm $Al_{0.33}Ga_{0.67}As$ barrier layer is deposited. Utilizing the local etching of Al droplets, symmetric nanoholes are formed and filled with 2.5 nm GaAs, overgrown with 69 nm $Al_{0.33}Ga_{0.67}As$ and capped with 4 nm of GaAs, producing a sample with strain-free GaAs QDs.

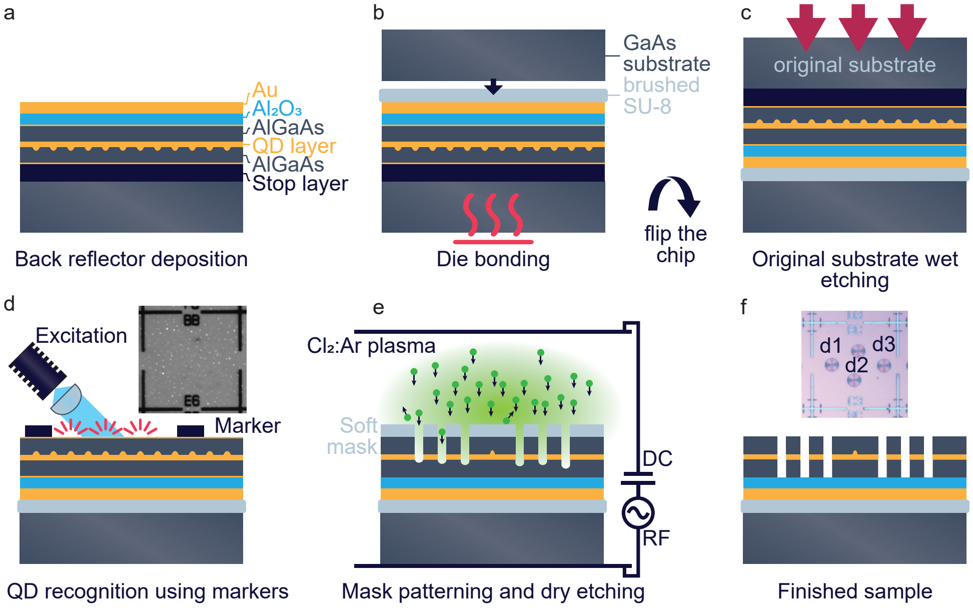

Figure S4: Sample fabrication, consisting of (a) the deposition of Al_2O_3 and Au, (b) bonding to a new substrate using SU-8, (c) multi-step wet-chemical etching of the original substrate, (d) cryogenic wide-field imaging and numeric image processing to map QD position, and (e) deterministic patterning and etching of CBRs, providing (f) a finished sample of QD-CBR devices.

A chart of the processing flow to obtain reflector-backed membranes is depicted in Figure S4(a-c). Pieces of approximately 10 mm^2 size are cut from the wafer and covered with 200 nm of Al_2O_3 by atomic layer deposition (ALD), a 2 nm layer of e-beam evaporated Cr and 150 nm of thermally evaporated Au, forming the back reflector of the cavity. Then, SU-8 2 is spread using a brush on the sample surface, dried and heated to 130 °C when the

sample is brought to contact with a same-size GaAs substrate. Crosslinking of polymers is initiated by applying a pressure of 2.2 MPa at a temperature of 230 °C for 10 min. Wet-chemical back-etching of the original substrate involves a fast etch system relying on the 3:7 mixture of 85 % H_3PO_4 and 30 % H_2O_2, stopping well before reaching the etch stop layer, and a 1:4 solution of 30 % H_2O_2 and powdered $C_6H_8O_7$ dissolved 1:1 in H_2O. The latter solution is selective between the substrate and etch stop material, leaving this layer to be removed with 10 % HF.

In order to introduce a frame of reference for recording QD positions, metallic reference markers are deposited on the surface of the back-etched sample. Electron-beam lithography (EBL) with 30 kV acceleration voltage is used to pattern marker crosses into the CSAR 62 e-beam resist, with an additional protective coating of Electra 92 to avoid charging effects. After the resist development, the sample is covered with a 150 nm stack of equally thick and strain-compensated Cr-Au-Cr layers. The lift-off in acetone and anisole reveals the marker fields defined by the remaining crosses and labels. To find QD positions, the sample is placed into a liquid He continuous-flow cryostat, the surface is illuminated with an infrared light-emitting diode (LED), whereas QDs are excited with a blue LED, making them visible through PL.[S5] Images of the sample with centered marker fields are processed numerically, to find QD positions, i.e., the center of fitted 2D-Gaussian shapes, with respect to the coordinate system, found by fitting the marker crosses. A sketch of the proceeding and an example of image can be seen in Figure S4(d).

To match the CBR spectrally with the QD emission, a set of empty resonators is etched into the sample and mode positions as a function of central disc radius r are characterized, as depicted in Figure S5(a). Since the spectral position of the exciton photon X is not recorded for single QDs but for the ensemble only, three designs d1, d2, and d3 are chosen to increase chances of spectrally matching the emission with the CM. A histogram of the emission statistics of this sample with the chosen target resonances at 782 nm (1.585 eV), 784 nm (1.581 eV), and 786 nm (1.577 eV) is provided in Figure S5(b). With known QD

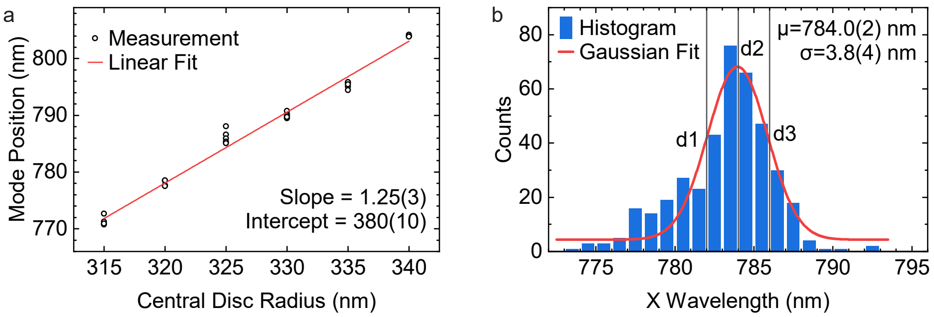

Figure S5: (a) Calibration curve to control the resonance of the CBR with the central disc radius r. (b) Histogram of the X wavelength fitted with a Gaussian with mean μ and standard deviation σ. Chosen CBR designs d1, d2, and d3 are marked.

positions and a calibrated r parameter, CBRs can be fabricated deterministically, using 100 kV EBL and reactive-ion etching with Cl_2:Ar chemistry in an inductively-coupled plasma reactor, yielding the finished sample, see Figure S4(e-f).

As mentioned in the main text, measurements indicate limited spectral and spatial overlap of the CBR with the embedded emitters. Due to a mistake in the design for deterministic EBL patterning, the actual realized parameters of the CBRs are periodicity $p = 380$ nm, trench width $t = 100$ nm, and radii $r_1 = 331$ nm, $r_2 = 332.6$ nm, $r_3 = 334.2$ nm, for designs d1, d2, and d3, resulting in cavities detuned red with respect to the QD emission. Furthermore, due to a rounding error in the script for detecting QD positions, QDs are off-center of the CBR, leading to polarized emission.[S6]

2.3 Optical Characterization

The sample is repeatedly measured between different etch cycles using optical characterization techniques in a confocal setup, sketched in Figure S6. A green 532 nm diode laser is used in continuous-wave (CW) operation to excite the QDs in the above-bandgap regime, whereas a wavelength-tunable pulsed titanium-sapphire (TiSa) laser enables on-demand photon generation from QDs (see below). Separate white light sources are used for widefield imaging and broadband reflectance measurements (see below). The polarization state of the light can be prepared with a polarizer (POL) and a rotating half-waveplate (HWP) and is directed to

the sample, residing in a liquid-He continuous-flow cryostat, as can be seen in Figure S6(b).

Polarization-resolved spectroscopy, imaging, as well as correlation and interferometry measurements can be performed plug-and-play by distributing the collected signal with single-mode (SM) fibers, flippable mirrors (flip-M), and flippable beam splitters (flip-BS) as depicted in Figure S6(a). To filter stray light from the room, a long-pass filter (LPF) is placed in front of the entrance slit of the spectrometer. The exit slit of the single [double] spectrometer enables it to be used as a monochromator, necessary for correlation experiments, depicted in Figure S6(c)[(d)]. Avalanche photodiodes (APDs) are used to detect single photons, connected to correlation electronics (see below). In Figure S6(e), a sketch of the Michelson interferometer shows a linear stage with a mounted retroreflector (RR) moving between interference visibility measurements using another RR on a piezoelectric actuator (see below).

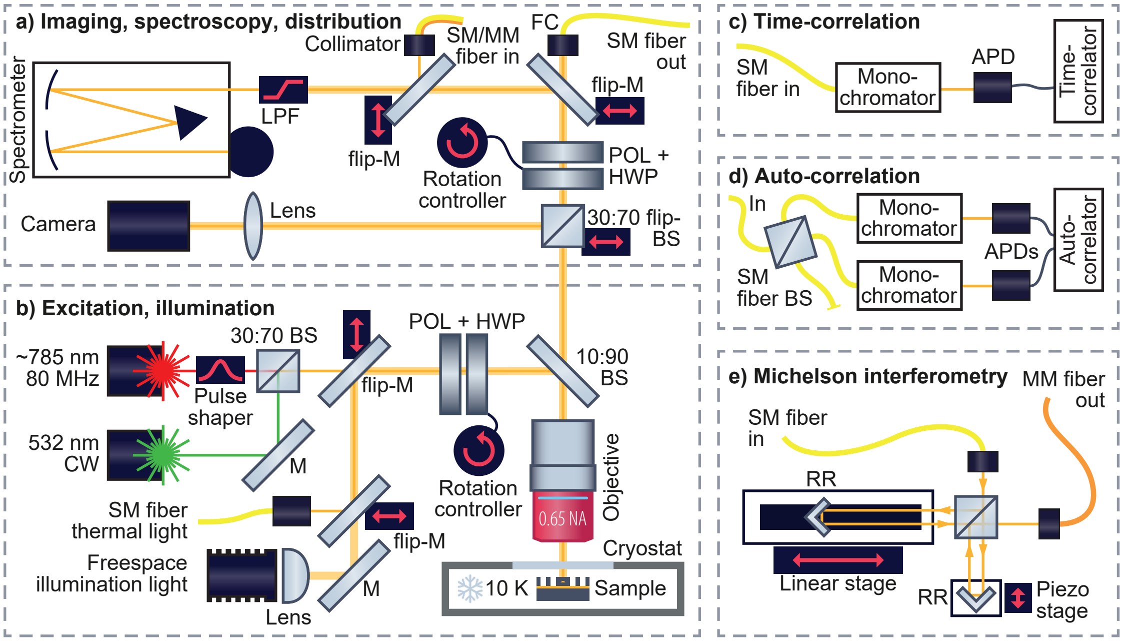

Figure S6: Sketch of the setup with highlighted dedicated areas.

2.3.1 Reflectance Measurements

Mode measurements of CBRs are conducted using reflectance measurements, where a thermal light from a halogen source is coupled into a SM fiber and directed to the sample. Reflected light is collected using a spectrometer with a 300 lines/mm diffraction grating, featuring a resolution of 0.21 nm, a vertical binning of 10 pixel (each 20×20 µm^2) of the charge-coupled device (CCD) to reduce noise contribution, and an entrance slit of 80 µm or larger. Obtained reflectance spectra are normalized by the reflected signal of the un-etched membrane surrounding the CBR structures. From experimental experience we observe that the reflectance minimum position is slightly dependent on the polarization (see above), as well as on the exact spatial position of the beam on the surface. Therefore, we conclude an experimental error of ± 1 nm of the mode position.

2.3.2 Two-Photon Excitation

In order to deterministically pump the biexciton state $|\mathrm{XX}\rangle$, two-photon-excitation (TPE) is used. To achieve this, a TiSa laser, producing pulses with a repetition rate of 80 MHz and a pulse length of about 100 fs, stretched to ∼5 ps using a pulse shaper, is employed. To excite QDs showing highly-polarized emission due to spatial misplacement with the cavity, it is necessary to match the polarization state of the excitation laser with the QD emission. The energy of the laser light is tuned to half of the energy E_{XX}, i.e., the distance between $|\mathrm{XX}\rangle$ and the ground state $|\mathrm{g}\rangle$. $|\mathrm{XX}\rangle$ shows a binding energy E_{b} with respect to the exciton state $|\mathrm{X}\rangle$:

$$E_{\mathrm{XX}}/2 = E_{\mathrm{X}} - E_{\mathrm{b}}/2 \tag{3}$$

For GaAs QDs this binding energy has found to be always positive and fairly constant at $E_b \approx 3.8$ meV(2 nm). Since $|\mathrm{XX}\rangle$ is driven resonantly, the state population experiences phonon-damped Rabi oscillations[S7,S8] as a function of laser power. All measurements are

conducted on the global maximum of the Rabi oscillations, the so called π-pulse.

2.3.3 Time-Correlation

Measurements of the lifetime of $|\mathrm{X}\rangle$ and $|\mathrm{XX}\rangle$ states are performed by time-correlated single photon counting. As mentioned before, emission into a polarized mode is favored on this sample; therefore, the polarization axis of the TPE pulse is aligned with respect to that axis. Furthermore, the emission is filtered with a rotated half-waveplate and a polarizer, such that only photons with a polarization state aligned to that axis are measured, as we expect the highest emitter–cavity coupling for this case. Emitted photons are collected using a SM fiber, filtered for X/XX signal using the exit slit of a spectrometer and forwarded to an APD with a time resolution of $\sim$ 100 ps, as depicted in Figure S6(c). To obtain more accurate results of lifetime values, the instrument response function (IRF) is recorded, by sending attenuated laser signal to the detector. For extracting the lifetimes of $|\mathrm{X}\rangle$ and $|\mathrm{XX}\rangle$ states, the histograms of the arrival time of the corresponding photons on an APD, relative to a clock from the pulsed excitation laser are fitted. Here we give a short explanation of the fitting functions.

From the rate equations, one can follow, that after the population of $|\mathrm{XX}\rangle$, there should follow an exponential decay from $|\mathrm{XX}\rangle$ to $|\mathrm{X}\rangle$ followed by a bi-exponential decay from $|\mathrm{X}\rangle$ to $|\mathrm{g}\rangle$. Therefore, the population as a function of time for the two states, corresponding to the time-dependent intensity profile $I_{\mathrm{X/XX}}(t)$ should have the following form:

$$I(t)_{\mathrm{XX}}(t) = A\exp\left(-\frac{t}{\tau_{\mathrm{XX}}}\right) \tag{4}$$

$$I(t)_{\mathrm{X}}(t) = B\exp\left(-\frac{t}{\tau_{\mathrm{X}}}\right) + C\exp\left(-\frac{t}{\tau_{\mathrm{XX}}}\right), \tag{5}$$

with τ_{XX} and τ_{X} being the lifetime of the XX/X respectively. The fitting function used to extract the lifetimes is the convolution of the exponential/bi-exponential decay with the

measured IRF.

2.3.4 Autocorrelation

The measurement of the second-order autocorrelation function $g^{(2)}(t)$ is realized in a Hanburry Brown-Twiss experiment. QD emission is coupled into a SM fiber, sent through a 50:50 fiber BS with the two outputs connected to two spectrometers used as monochromators, and forwarded to APDs, connected with correlation electronics, as sketched in Figure S6(d). In the obtained correlation histogram, as seen in the main text, coincidences of detection events on both detectors are plotted as a function of the time delay between the detection of those two photons. The antibunching nature of single photons reveals a dip at 0-time delay, indicating a reduced probability of detecting two photons simultaneously. The coincidence peaks of photons originating from different excitation cycles are separated by 12.5 ns corresponding to the 80 MHz repetition rate of the excitation laser.

The second-order autocorrelation function is evaluated at 0-time delay, by integrating 2 ns around the 0-time delay dip divided by the mean of the two closest neighboring peaks, also integrated 2 ns. Further side peaks are neglected, due to fast blinking dynamics of the QD (telegraph noise). A method to reduce blinking is to assist the TPE with a green CW laser to maximize XX photon brightness; however, this degrades the $g^2(0)$ value of X photons, due to non-resonant undesirable excitation events of $|\mathrm{X}\rangle$.

2.3.5 Michelson Interferometry

Michelson interferometry, i.e., the measurement of the first-order coherence $g^{(1)}(t)$, is used to determine the coherence properties of a light source, and thereby, further quantify its optical quality. The main components of the interferometer are a 50:50 BS, a retroreflector (RR) on a linear stage and a RR on a piezo stage. The incoming beam is split into two ideally identical parts, which are then reflected back onto the BS to interfere there. The piezo performs several steps of 20 nm for each position on the linear stage. There, the step

size is chosen according to the expected coherence length in the range of a few mm. A sketch of a Michelson interferometer is given in Figure S6(e). The intensity of one of the outputs of the BS is measured using a spectrometer. For each position of the linear stage, the intensity of the emission line of interest is integrated which leads to sets of intensity data where the interference is visible for different relative delay times. These are fitted using a cosine function, to extract the visibility ν at each of a given time delay:

$$\nu = \frac{I_{max} - I_{min}}{I_{max} + I_{min}} \tag{6}$$

This is valid, if we assume that the optical path difference created by the piezo stage is very small compared to the coherence length and therefore the visibility is constant in that range. This visibility in dependence of the relative delay time is fitted using the Fourier-transform (FT) of a Voigt function, which we explain now in more detail:

The lower limit on the radiative line width is given by the lifetime $\tau_{\mathrm{X/XX}}$ of the exponential decay of $|\mathrm{X}\rangle$ and $|\mathrm{XX}\rangle$,leading to a Lorentzian line shape with a FWHM of $\Gamma_{0,\mathrm{X/XX}} = \hbar/\tau_{\mathrm{X/XX}}$, which is called the natural line width. QD emission lines are broadened by spectral wandering, which occurs when there are charge fluctuations in the vicinity of the QD.[S9,S10] This leads to a Gaussian broadening on macroscopic timescales, therefore in a Michelson measurement, the line width measured is the convolution of both effects:

$$V(E,\sigma,\gamma) = (G * L)(E) = \int G(\epsilon)L(\epsilon - E)d\epsilon, \tag{7}$$

$$G(E,\sigma) = \frac{1}{\sigma\sqrt{2\pi}}e^{-\frac{E^2}{2\sigma^2}}, \tag{8}$$

$$L(E,\gamma) = \frac{\gamma}{\pi\left(E^2 + \gamma^2\right)}, \tag{9}$$

with the Voigt profile $V(E)$, the Gaussian profile $G(E)$, and the Lorentzian profile $L(E)$

as function of photon energy E, and widths of the distributions σ and γ. The FT, which transforms the energy profile into the time profile where the Michelson measurement takes place, simplifies this to a multiplication, which is used for fitting the visibility data:

$$\mathrm{FT}[V(E,\sigma,\gamma)] = \hat{V}(t,t_G,t_L) = \hat{G}(t,t_G)\cdot\hat{L}(t,t_L), \tag{10}$$

with the coherence-times t_G and t_L for the Gaussian, and the Lorentzian part, respectively, and $\hat{V}$, $\hat{G}$, and $\hat{L}$, the FT of the corresponding profiles. The widths σ,γ in the energy picture can be calculated using:

$$\sigma = \frac{\hbar}{t_G} \quad \text{and} \quad \gamma = \frac{\hbar}{t_L}. \tag{11}$$

In order to calculate the FWHM f_V of the Voigt profile, the FWHM values $f_{G,L}$ are needed:

$$f_G = 2\sigma\sqrt{2\ln 2} \tag{12}$$

$$f_L = 2\gamma \tag{13}$$

Since the Voigt profile does not have an analytical form, the width can not be calculated analytically but there is an approximation:[S11]

$$f_V \approx 0.5346 f_L + \sqrt{0.2166 f_L^2 + f_G^2} \tag{14}$$

For a purely Gaussian line, this approximation fits perfectly, while for an arbitrary Voigt profile it produces results with an accuracy of around 0.02%. Widths f_V correspond to the measured line widths provided in the main text.

References

(S1) Mosor, S.; Hendrickson, J.; Richards, B. C.; Sweet, J.; Khitrova, G.; Gibbs, H. M.; Yoshie, T.; Scherer, A.; Shchekin, O. B.; Deppe, D. G. Scanning a photonic crystal slab nanocavity by condensation of xenon. *Applied Physics Letters* **2005**, *87*, 1–3.

(S2) Johnson, P. B.; Christy, R.-W. Optical constants of the noble metals. *Physical review B* **1972**, *6*, 4370.

(S3) Huang, H.; Manna, S.; Schimpf, C.; Reindl, M.; Yuan, X.; Zhang, Y.; da Silva, S. F. C.; Rastelli, A. Bright Single Photon Emission from Quantum Dots Embedded in a Broadband Planar Optical Antenna. *Advanced Optical Materials* **2021**, *9*, 2001490.

(S4) Novotny, L.; Hecht, B. *Principles of Nano-Optics*, 2nd ed.; Cambridge University Press, 2012; p 224–281.

(S5) Krieger, T. M.; Rota, M. B.; Freund, J.; Covre da Silva, S. F.; Manna, S.; Trotta, R.; Rastelli, A. Diffraction-Limited Cryogenic Imaging of Quantum Emitters for Deterministic Photonic Integration. Optica Quantum 2.0 Converence and Exhibition. 2023; p QW2A.36.

(S6) Peniakov, G.; Buchinger, Q.; Helal, M.; Simon, B.; Reum, Y.; Rota, M. B.; Ronco, G.; Beccaceci, M.; Krieger, T. M.; Covre da Silva, S. F.; Rastelli, A.; Trotta, R.; Pfenning, A.; Huber-Loyola, T.; Höfling, S. Polarized and Un-Polarized Emission from a Single Emitter in a Bullseye Resonator. *Manuscript in preparation* **2023**,

(S7) Förstner, J.; Weber, C.; Danckwerts, J.; Knorr, A. Phonon-Assisted Damping of Rabi Oscillations in Semiconductor Quantum Dots. *Phys. Rev. Lett.* **2003**, *91*, 127401.

(S8) Stufler, S.; Machnikowski, P.; Ester, P.; Bichler, M.; Axt, V. M.; Kuhn, T.; Zrenner, A. Two-photon Rabi oscillations in a single $In_xGa_{1-x}As$/GaAs quantum dot. *Phys. Rev. B* **2006**, *73*, 125304.

(S9) Kamada, H.; Kutsuwa, T. Broadening of single quantum dot exciton luminescence spectra due to interaction with randomly fluctuating environmental charges. *Physical Review B - Condensed Matter and Materials Physics* **2008**, *78*, 1–16.

(S10) Kuhlmann, A. V.; Houel, J.; Ludwig, A.; Greuter, L.; Reuter, D.; Wieck, A. D.; Poggio, M.; Warburton, R. J. Charge noise and spin noise in a semiconductor quantum device. *Nature Physics* **2013**, *9*, 570–575.

(S11) Olivero, J. J.; Longbothum, R. L. Empirical fits to the Voigt line width: A brief review. *Journal of Quantitative Spectroscopy and Radiative Transfer* **1977**, *17*, 233–236.
